# Sperm quality parameters of Swiss army recruits pre- and peri-COVID-19: a cross-sectional, comparative analysis

**DOI:** 10.1016/j.nmni.2026.101761

**Published:** 2026-05-05

**Authors:** Sabine Köppen, Ulrike Held, Nejla Gültekin, Andreas Stettbacher, Zeno Stanga, Ismail I. Ülgür, Jeremy Werner Deuel, Patricia Schlagenhauf

**Affiliations:** a Department of Global and Public Health, Epidemiology, Biostatistics and Prevention Institute, University of Zurich, Zurich, Switzerland; b Department of Biostatistics, Epidemiology, Biostatistics and Prevention Institute, University of Zurich, Zurich, Switzerland; c Centre of Competence for Military and Disaster Medicine, Swiss Armed Forces, Ittigen, Bern, Switzerland; d Division of Medical Oncology and Haematology, University Hospital of Zurich, Zurich, Switzerland; e WHO Collaborating Centre for Travellers' Health, Department of Global and Public Health, MilMedBiol Competence Centre, University of Zurich, Zurich, Switzerland

**Keywords:** Male fertility, Sperm quality, Young men, Geographical differences, Reproductive hormones

## Abstract

**Background:**

This study evaluates sperm quality among young Swiss military recruits in 2021 and compares the findings with data collected between 2005 and 2017, in the context of the reported global decline in sperm quality.

**Methods:**

This cross-sectional study compares two cohorts of Swiss men aged 18 to 30 years. Group 1 (Rahban) included 2523 participants examined between 2005 and 2017, and Group 2 (LoCoMo) comprised 194 recruits assessed in 2021. Semen analyses were performed according to WHO guidelines. In Group 2, reproductive hormones (FSH, LH, testosterone) were additionally measured. Comparisons between cohorts were conducted, and geographic variation within Group 2 was assessed by language region and settlement type.

**Results:**

Based on WHO reference criteria, 17% of soldiers of Group 2 (LoCoMo in the peri-COVID period) had oligozoospermia, 35% had asthenozoospermia, and 14% teratozoospermia. Compared to Group 1 (Rahban), sperm concentration, total sperm count, progressive sperm motility and sperm morphology remained similar. Evidence for differences in LH and FSH levels of Group 2 were found in three different sperm concentration groups, while no evidence of differences were shown in testosterone levels. No evidence of differences in distribution of sperm concentration groups were found between language regions (Latin vs. Non-Latin) and settlement types ((Sub)urban vs. Rural).

**Conclusions:**

Sperm quality remained stable in 2021 apart from those with recent COVID-19 infections compared to the period from 2005 to 2017. Larger studies are required to explore spatial correlation of sperm parameters and other influencing factors.

## Teaser

A Swiss study on military recruits compared sperm quality from 2005 to 2017 to more recent data from 2021, showing that sperm quality has remained stable. Geographic analyses revealed no evidence for differences in sperm quality according to language regions and urban versus rural area of residence.

## 1 Introduction

Infertility is the complete inability to conceive, while subfertility refers to difficulty conceiving after at least one year of unprotected intercourse [1,2]. Sperm quality is a central indicator of male fertility [3]. Sperm quality is assessed based on semen volume, sperm concentration, progressive sperm motility, and sperm morphology, according to reference criteria of the World Health Organization (WHO) [4]. Recent retrospective data (2021–2022) suggest that total motile sperm count is a superior predictor of biochemical pregnancy, clinical pregnancy, and live birth rates. Accordingly, this parameter has been included in the present analysis [5].

Many studies have reported a decline in sperm quality over the past decades, including Carlsen et al. in a review of 61 papers in 1992, which confirmed a decline in sperm quality, particularly in mean sperm count, semen volume and sperm density over the last 50 years [6]. A meta-regression analysis published in 2017 has reported a significant decline of 50–60% in sperm counts between 1973 and 2011 among men from ‘Western’ regions including North America, Europe, Australia and New Zealand versus ‘Other’ regions including South America, Asia and Africa [7]. A subsequent meta-analysis showed that the sperm count is also declining at an accelerated rate in South/Central America-Asia-Africa regions (percent decline per year of 1.16% post–1972 to 2.64% post–2000) [8]. This development represents a significant challenge for public health and requires a deep understanding of the underlying causes and contributing factors.

One such factor is the level of sex hormones, whose impact on sperm quality has been investigated in numerous studies. For example, a study from 2006 involving 75 men with fertility issues, defined as one year of unexplained childlessness after ruling out any obvious issues in the female partner, found that LH and FSH levels were inversely correlated with sperm count, motility, and morphology, while testosterone showed no significant correlation [9]. Conversely, Meeker et al. observed an inverse correlation between FSH and LH levels with sperm concentration, motility, and morphology, but a positive correlation between testosterone levels and sperm motility [10]. More recently, a study of 338 subfertile men reported that LH, FSH and Testosteron levels were inversely associated with sperm progressive motility while only LH and FSH were inversely associated with sperm morphology [11]. In addition to hormonal factors, geographical differences in sperm quality offer valuable insights into genetic, epigenetic, environmental and lifestyle influences on reproductive health [12,13]. Several studies investigated sperm quality with a focus on environmental factors, in particularly the differences between urban and rural areas [14]. In 2003, Swan et al. evaluated differences in four different urbanization areas in the US (Missouri, New York, Minnesota, and California). Reduced sperm concentration and motility were observed in semirural and agricultural areas compared to more urban and less agriculturally exposed areas [15]. A French study has reported a decrease in sperm concentration and morphology. It was remarkable that the most significant decreases and lowest values were found in two neighboring regions that are both heavily agricultural and densely populated [16]. A study population consisted of 1291 young men from four different regions in Russia were divided into the three largest ethnic groups. Significant regional and ethnic differences in sperm and reproductive hormone parameters were observed, suggesting that ethnic and environmental factors influence regional differences [17].

While many studies have compared male fertility across different countries, there is a lack of studies focusing on variations of sperm parameters within a single country. In Switzerland, Rahban et al. evaluated sperm quality for the period 2005–2017. The study involved a group of 2523 young men aged between 18 and 22 assessed at six different recruitment centers. The study was based on 2523 samples out of total of 92,274 conscripts being present in the conscription centers between 2005 and 2017. According to WHO semen reference criteria, 62% of Swiss men, were found to have suboptimal sperm quality, defined as at least one parameter below the WHO thresholds. No major differences of sperm parameters in geographic, linguistic and language regions were identified [18].

This current study aims to assess sperm quality among young Swiss army soldiers in 2021, using samples collated during the COVID-19 pandemic in the framework of the Long COVID in Military Organization (LoCoMo) study [19]. These samples were part of a study to assess the multisystem consequences of SARS-CoV-2 infection in young men, which found evidence for poorer progressive motile sperm count in the subgroup of recent COVID-19 infections (≤180 days before testing) [19].

In this analysis we focused on hormonal correlations with male fertility, the impact of a SARS-CoV-2 infection and we geographically analyzed sperm quality in Switzerland by settlement type (Sub(urban) vs. Rural) and linguistic (Latin vs. Non-Latin) stratification. Additionally, the study sought to compare sperm quality parameters for the periods 2005–2017 (Group 1, Rahban) [18] with those from 2021 (Group 2, LoCoMo) [19].

## 2 Methods

This study compares sperm quality parameters in two groups of subjects [18]. The first group of 2523 men from the military recruitment days were assessed for sperm quality once in the period 2005 to 2017 and the results were published [18].

The second group of Swiss military soldiers were recruited in the framework of the LoCoMo study which evaluated sequelae of SARS-CoV-2 infection on a number of health parameters including sperm quality in the year 2021 [19].

This study uses data from two previously published studies, both of which received Swiss ethical committee approval. The two studies in question are: Rahban [18] and LoCoMo [19].

### 2.1 Study population - group 1 (Rahban)

Data were collected from recruits between the ages of 18 and 22 once in the period from September 2005 to June 2017. The survey was conducted in six recruitment centers in Switzerland (Lausanne, Windisch, Monteceneri, Rüti, Sumiswald, Mels). There were no exclusion criteria during the data collection. Ejaculation abstinence was recommended for two days [18].

#### 2.1.1 Questionnaires and clinical data – group 1 (Rahban)

The participants filled out a questionnaire on personal details (age, height, weight, diagnosed diseases (including urogenital diseases), lifestyle (smoking, alcohol consumption, and education) were called for a physical examination, and a sperm sample was provided. The basic sperm parameters (semen volume, total sperm count, sperm concentration, sperm motility, sperm morphology) were evaluated [18].

### 2.2 Study population - group 2 (LoCoMo)

Data on health and sperm quality were collected from soldiers aged between 18 and 30 years in Switzerland. Inclusion criteria for participation was a positive or negative reverse transcription polymerase chain reaction (RT-PCR) test for SARS-CoV-2 during their army service between March 2020, and December 2020 and no known male reproductive anomalies [19]. Participant evaluation occurred once in the period from May 2021 to November 2021. For this study, we included 194 soldiers of the 241 agreeing to have their sperm analyzed. Participants were excluded mostly for not adhering to the required 2–7 days sex abstinence period (10%) or missing data (9.5%) [20].

#### 2.2.1 Questionnaires and clinical data – group 2 (LoCoMo)

Data from the military survey was used to assess male fertility and possible influencing factors. These included information on general health (age, height, weight) and lifestyle (smoking, alcohol consumption, frequency of physical activity). Genital anomalies, such as cryptorchidism and varicocele, were asked about. Sex hormones (follicle-stimulating hormone (FSH) and luteinizing hormone (LH), total testosterone) were measured. To assess sperm quality, the basic sperm parameters (pH, semen volume, total sperm count, sperm concentration, progressive sperm motility and sperm morphology) as well as the total motile sperm count were determined in the ejaculate. The period of ejaculation abstinence was also recorded.

### 2.3 Statistical analysis

All statistical analyses were conducted using R software, version 4.3.3.

#### 2.3.1 Sperm analysis - group 2 (LoCoMo)

To ensure comparability, we evaluated fertility parameters of Group 2 (LoCoMo) according to the methodology used in the study of Group 1 (Rahban) [18]. For continuous variables, the median was calculated alongside the interquartile range (25th and 75th percentiles). For categorical variables, frequencies and corresponding percentages were calculated.

In the multivariable analysis of Group 2 (LoCoMo), data were evaluated for different categories:

• Total population: the entire study population (n = 194)
• Control subgroup: Soldiers without a history of COVID-19 infection (n = 87). Previous studies have reported a negative impact of COVID-19 on sperm quality. This subgroup was created to control for this potential confounding effect [19,21], and
• Sperm concentration group I, II, III: total population (n = 194) stratified into three groups based on WHO reference thresholds for sperm concentration applied across all variables of interest to enable categorical comparisons.

Sperm concentration was categorized into three groups (I, II, III): I) < 15 Mio/mL, II) 15–40 Mio/mL, and III) > 40 Mio/mL. The lower threshold of 15 Mio/mL corresponds to the 5th percentile threshold for male fertility, as defined by the WHO [4]. The upper threshold of 40 Mio/mL corresponds with the ‘time to pregnancy’ (TTP) threshold, which denotes that TTP increases with sperm concentration up to 40–50 Mio/mL. TTP refers to the number of months required for a couple to achieve pregnancy when not using any methods to avoid pregnancy [22]. In addition to the basic sperm quality parameters, we analyzed the total motile sperm count, which has been shown to better predict male fertility, particularly regarding pregnancy outcomes in couples undergoing intracytoplasmic sperm injection [23]. For the analysis, the Pearson correlation coefficient with the Bonferroni correction for multiple testing was used for the continuous parameters and the Cochrane-Armitage trend test with the Benjamini-Hochberg correction for the categorical parameters. The p-value was calculated to evaluate differences among the three groups, with statistical significance set at p < 0.05.

#### 2.3.2 Comparison of sperm parameters – group 1 (Rahban) vs. group 2 (LoCoMo)

Sperm parameters of the Rahban (Group 1) and LoCoMo (Group 2) study were compared by medians and the 5th to 95th percentiles [18]. Given that the LoCoMo study was designed to assess COVID-19 related effects, data analysis was further subdivided into the following groups: the recent COVID-19 group (≤180 days since positive PCR test), the non-recent COVID-19 group (>180 days since a positive PCR test), the asymptomatic infection group (serologically positive but with no symptoms) and the control group (serologically negative) [18]. Comparisons were made between Group 1 (Rahban: total population) and four different (sub)groups of Group 2: LoCoMo (total population) and three subgroups (LoCoMo recent COVID-19 subgroup, LoCoMo non-recent/asymptomatic COVID-19 subgroup, LoCoMo control subgroup). In each case, the median and the 5th to 95th percentiles were calculated.

**Fig. 1:**
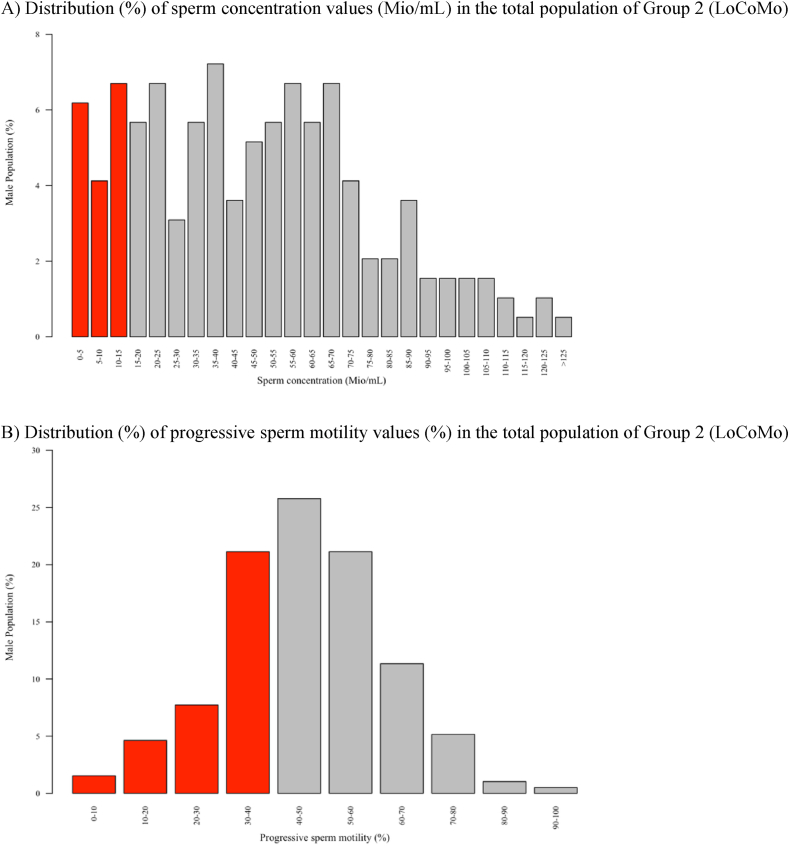

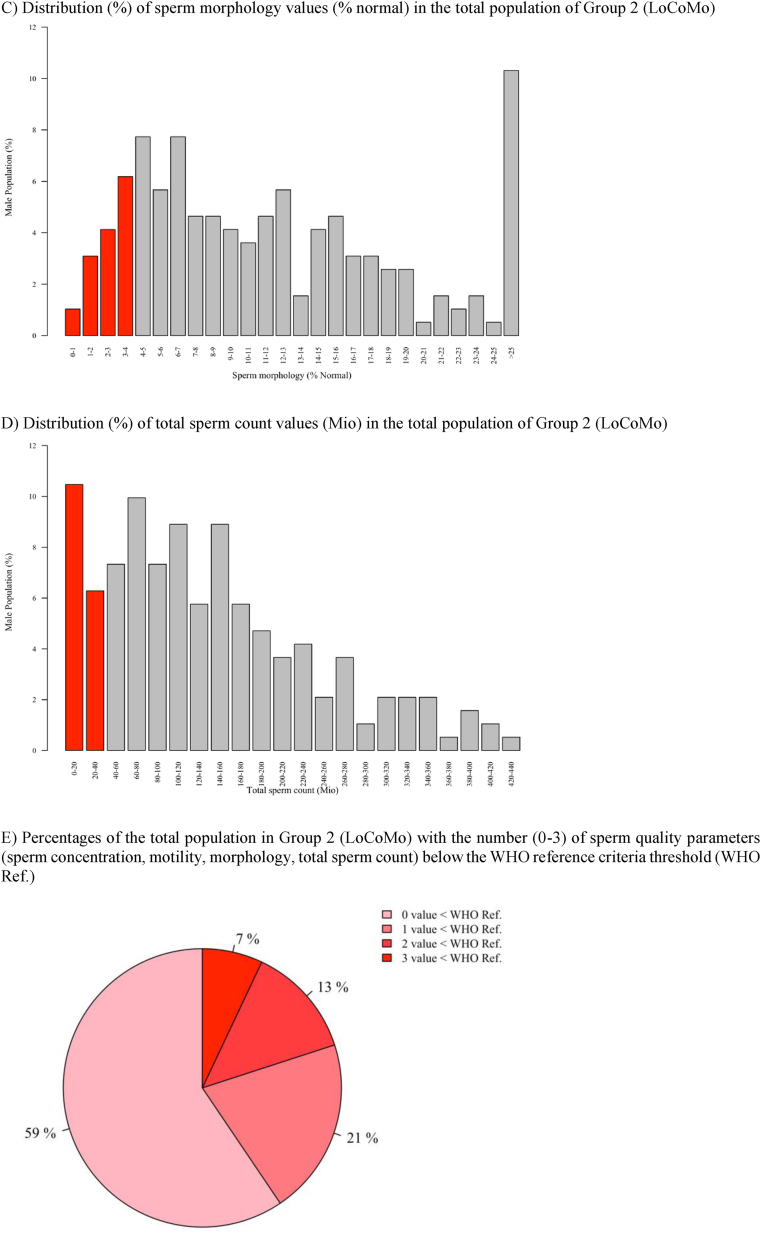
**Visualization of Sperm Parameter Distributions – Group 2 (LoCoMo).** The bar charts illustrate the distribution of the frequencies of basic sperm quality parameters of Swiss army recruits (n = 194). A) 17% of the cases have oligozoospermia with a sperm concentration in the ejaculate below the WHO threshold value of 15 Mio/mL. B) 17% of the cases have a total sperm count below 40 Mio in their ejaculate. C) 35% have asthenozoospermia with less than 40% motile sperm. D) 14% have teratozoospermia with less than 4% morphologically normal sperm. E) Representation of a pie chart with proportions over the number of parameters with values below the WHO threshold; 41% of men have at least one value below the thresholds set for fertile men. Red columns illustrate the percentage of men with a value below the WHO threshold.

For the calculation of the Wilcoxon rank-sum test, the sperm motility data of Group 1 (Rahban) were extracted from Fig. 1C using Plot Digitizer and subsequently analyzed [24].

### 2.4 Geographical analysis – group 2 (LoCoMo)

For the geographical representation of the sperm concentration of Group 2 (LoCoMo), postal code information was used from the military survey. The postal codes were georeferenced with the Federal Statistical Office (FSO) numbers from Swisstopo in the open source Quantum Geographic Information System (QGIS) version 3.38 [23,25]. For the cartographic representation of the sperm concentration, the data set was imported into the program R, version 4.3.3, and geographical data on national boundaries from the FSO were utilized [25,26].

The data was divided into four language regions of Switzerland: German, French, Italian and Romansh [27]. For the analysis, language regions were grouped into “Latin” (French, Italian, and Romansh) and “Non-Latin” (German). To assess whether the distribution of sperm concentration categories differed between language regions, a Cochran-Armitage trend test was performed, evaluating whether there was an evident trend in the sperm concentration groups (0–15 Mio/mL, 15–40 Mio/mL, and >40 Mio/mL) across the two language region groups (Latin vs. Non-Latin).

In addition, a further stratification was made according to the residence: urban, suburban and rural [28]. A Cochran-Armitage trend test was used to assess whether sperm concentration categories were distributed differently between (sub)urban and rural regions.

## 3 Results

### 3.1 Description of the study population – group 2 (LoCoMo)

Group 2 (LoCoMo) included a total of 194 young Swiss soldiers. The baseline characteristics and sperm parameters of the study population are presented in Table 1. The majority of participants were healthy and had no reproductive abnormalities, such as varicocele or cryptorchidism (Table 1; C). General characteristics, lifestyle factors, including alcohol consumption, smoking, and frequency of sport, did not show significant differences between the concentration groups I, II and III (Table 1; B).

**Table 1:** **Multivariable description of Group 2 (LoCoMo) including basic sperm quality parameters.** Analysis of the total population, the control subgroup, and subdivision into sperm concentration groups (I, II, III). The continuous parameters were calculated using the median and the interquartile range (25th and 75th percentile) and the categorical parameters as frequencies and the percentages.

	N with data	Total Population (n = 194)		Control subgroup (n = 87)	
**A: General characteristics**					
Age (years)	194	22	(21⎼23)	21	(20⎼22)
Height (cm)	194	180	(176⎼184)	180	(177⎼184)
Weight (kg)	194	76	(70⎼85)	75	(70⎼81)
BMI (kg/m^2^)^a^	194	23.7	(22⎼25)	22.9	(22⎼24)
**B: Lifestyle factors**					
Cigarettes smokers (%)^b^	193	43	(22%)	21	(24%)
Smoking (years), smokers only	71	3	(2⎼6)	4	(2⎼6)
Cigarettes/day, smokers only	70	5.5	(2⎼10)	5	(1⎼10)
Alcohol consumers (%)^c^	193	176	(91%)	79	(91%)
Alcohol, consumers only (units/week)	176	3	(2⎼6)	4	(2⎼6)
Sport frequency/week (30min)^d^	194	2	(1⎼4)	2	(1⎼4)
**C: Genital Anomalies**					
Cryptorchidism treated (%)	192	3	(1.6%)	2	(2.3%)
Varicocele operated (%)	192	3	(1.6%)	2	(2.3%)
**D: Sex hormones**					
LH (mIU/mL)	191	5.2	(4.3⎼6.4)	5.2	(4⎼6)
FSH (mIU/mL)	190	3.2	(2.2⎼4.5)	3.1	(2⎼4)
Total Testosterone (nmol/L)	178	20.1	(16.3⎼23.4)	20.8	(17⎼25)
**E: Semen parameters**					
Ejaculation abstinence (days)	194	4	(3⎼4)	3	(3⎼4)
pH	192	7.7	(7.5⎼7.7)	7.7	(7.5⎼7.7)
Volume (mL)	194	3	(2⎼4.3)	2.8	(2⎼4.4)
Sperm Concentration (Mio/mL)	194	45	(23⎼65)	50	(23⎼65)
Total sperm count (Mio)	194	120	(64.5⎼200)	119	(59.3⎼200.8)
Progressive sperm motility (%)	194	46	(35.3⎼56)	45	(35.5⎼54.5)
Total progressive sperm count (Mio)	194	56	(21.2⎼106.4)	53.2	(20.4⎼96.4)
Normal morphology (%)	194	10	(5⎼16)	11	(5⎼16.5)

	Sperm Concentration (Mio/mL)						P-value^e^
	Group I: <15 (n = 34)		Group II: 15-40 (n = 59)		Group III: >40 (n = 101)		
**A: General characteristics**							
Age (years)	21	(20⎼22)	22	(21⎼23)	22	(20⎼23)	0.43
Height (cm)	183	(179⎼188)	180	(176⎼184)	180	(176⎼184)	0.19
Weight (kg)	82	(73⎼95)	77	(67⎼85)	76	(70⎼81)	0.20
BMI (kg/m^2^)^a^	24	(23⎼27)	23	(22⎼25)	23.9	(22⎼25)	0.47
**B: Lifestyle factors**							
Cigarettes smokers (%)^b^	12	(35%)	12	(21%)	19	(19%)	0.29
Smoking (years), smokers	4	(3⎼6)	3	(2⎼7)	3	(2⎼6)	0.79
Cigarettes/day, smokers	5	(2⎼10)	10	(4⎼10)	5	(1⎼10)	0.79
Alcohol consumers (%)^c^	31	(91%)	52	(90%)	93	(92%)	1.00
Alcohol, consumers only (units/week)	5	(3⎼9)	3	(1⎼5)	3	(2⎼6)	0.14
Sport frequency/week (30min)^d^	2	(1⎼2)	2	(1⎼4)	2	(1⎼4)	0.79
**C: Genital Anomalies**							
Cryptorchidism treated (%)	1	(2∙9%)	2	(3∙4%)	0	(0%)	0.47
Varicocele operated (%)	0	(0%)	0	(0%)	3	(3%)	0.53
**D: Sex hormones**							
LH (mIU/mL)	5.9	(5⎼7)	5.7	(5⎼7)	4.8	(4⎼6)	**<0.001**
FSH (mIU/mL)	4.6	(3⎼6)	3.5	(3⎼5)	2.8	(2⎼4)	**<0.001**
Total Testosterone (nmol/L)	20	(17⎼23)	20.5	(16⎼24)	19.5	(16⎼23)	0.63
**E: Semen parameters**							
Ejaculation abstinence (days)	3.5	(3⎼4)	4	(3⎼4)	4	(3⎼5)	0.31
pH	7.7	(7.5⎼7.8)	7.7	(7.5⎼7.7)	7.7	(7.5⎼7.7)	0.43
Volume (mL)	3	(2.3⎼4.8)	3.3	(2⎼4.6)	2.5	(2⎼4)	0.35
Sperm Concentration (Mio/mL)	7	(3.6⎼11)	30	(23⎼35)	65	(55⎼83)	⏤
Total sperm count (Mio)	20.4	(10⎼42)	80	(50.9⎼140.3)	189.8	(120⎼275)	⏤
Progressive sperm motility (%)	33	(24.5⎼41.5)	45	(34⎼55.5)	50	(40⎼59)	⏤
Total motile sperm count (Mio)	6.75	(2.8⎼15.1)	38.1	(20.1⎼60.1)	90	(58.2⎼141.5)	⏤
Normal morphology (%)	4	(2⎼6.8)	8	(5⎼14.5)	12	(8⎼18)	⏤

a BMI = Body Mass Index.

b Cigarette smokers includes all study participants who smoked regularly or occasionally according to the survey.

c Alcohol consumers includes all study participants who drink alcohol regularly or occasionally according to the survey.

d In the survey for training frequency per week, the answers <1, 1, 2, 3-4x, almost daily were available. The numbers of training sessions per week were approximated as 0, 1, 2, 3.5, 6.

e The p-value was calculated for the comparison of the parameters in the groups. The Pearsons-Correlation test with the Bonferroni correction for multiple testing was used for the continuous parameters and the Cochrane-Armitage trend test with the Benjamini-Hochberg correction for the categorical parameters. A p-value below 0.05 was considered statistically significant and was highlighted in bold.

Evidence for an increase in LH and FSH levels was observed with decreasing sperm concentration, while testosterone levels did not show any statistically significant differences (Table 1; D).

### 3.2 Visualization of sperm quality - group 2 (LoCoMo)

Fig. 1 illustrates the distribution of sperm parameter values of Group 2 (LoCoMo) using bar charts.

Based on WHO guidelines for male fertility, 17% of the participating recruits had a sperm concentration of less than 15 Mio/mL (Fig. 1; A), and 17% had a total sperm count of less than 40 Mio per ejaculate (Fig. 1; B). Additionally, 35% of the recruits exhibited progressive sperm motility of less than 40% (Fig. 1; C), while 14% had normal morphology in fewer than 4% of sperm (Fig. 1; D). Overall, 41% of the recruits had at least one sperm parameter below the WHO threshold for male fertility (Fig. 1; E) [20].

### 3.3 Comparison of the distribution of sperm parameters – group 1 (Rahban) vs. group 2 (LoCoMo)

Compared to the distribution of sperm parameter values in the Rahban et al. study (Group 1), the frequency of sperm concentrations and the total number of sperms below the threshold were very similar (both 17%). Compared to the LoCoMo study (Group 2), Group 1 (Rahban) had a lower percentage of values below the WHO threshold for progressive sperm motility (25%) and a higher percentage for sperm morphology (43%). In Group 1 (Rahban) 62% and in Group 2 (LoCoMo) 41% of men had at least one value below the thresholds set for fertile men [18].

Based on the comparison of the bar charts, a decrease in sperm motility from Group 1 (Rahban) to Group 2 (LoCoMo) could be assumed. To statistically support this hypothesis, a Wilcoxon rank-sum test was performed which showed no evidence for significance: W = 47, p = 0.49 [18].

### 3.4 Comparison of sperm parameters – group 1 (Rahban) vs. group 2 (LoCoMo)

**Fig. 2:**
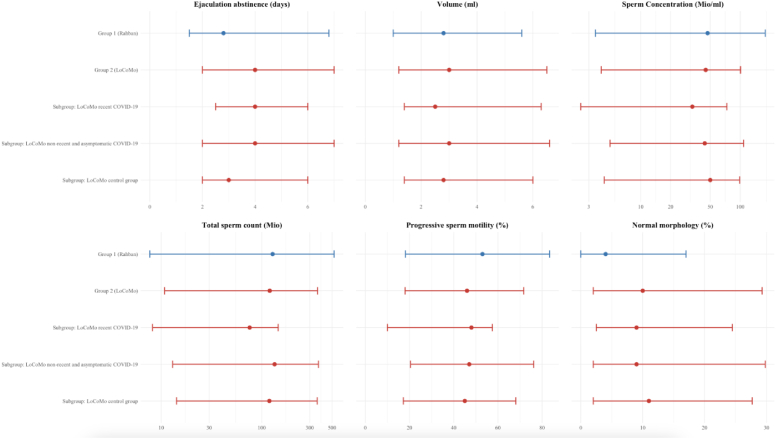
Comparison of sperm quality parameters: Group 1 (Rahban) and Group 2 (LoCoMo). Comparison of six different sperm parameters between Group 1 (Rahban) (n = 2523) and the Group 2 (LoCoMo) (n = 194), including its subgroups (LoCoMo recent COVID-19 (n = 11), LoCoMo non-recent/asymptomatic COVID-19 (n = 96) and LoCoMo control group – no COVID-19 infection (n = 87)). Each dot represents the median value of a parameter, with horizontal lines indicating the 5th to 95th percentile. The axes are separately scaled for each parameter. The axes of the parameters sperm concentration and total sperm count are logarithmically scaled.

Group 1 (Rahban) and 2 (LoCoMo) were listed in Fig. 2 for comparability. The range of ejaculation abstinence in Group 1 (Rahban) was broader due to differing study conditions. For the total population of Group 1 (Rahban) and 2 (LoCoMo), the 5th to 95th percentile for every parameter overlap.

By comparing the subgroups, it was found that the recent COVID-19 subgroup had the lowest sperm concentration as well as the lowest total sperm count when comparing the median values suggesting a transient impact of COVID-19 infection on sperm quality.

### 3.5 Geographical analysis of group 2 (LoCoMo): stratification of men according to language regions and area of residence

The Non-Latin (German-speaking) region had the highest number of recruits in the sample. In both the Latin and Non-Latin regions, the highest sperm concentration category (>40 Mio/mL) included the largest number of recruits (Table 2).

**Table 2:** Geographical analysis of sperm concentration groups in young Swiss recruits. An analysis of linguistic and area of residence variations in recruits of Group 2 (LoCoMo): A) Distribution of sperm concentration (in Mio/mL) among recruits by language region (Latin vs. Non-Latin). B) Distribution of sperm concentration (in Mio/mL) among recruits by area of residence ((Sub)urban vs. Rural). Values are presented as absolute frequencies (number of recruits) and percentages (total n = 194).

A) Language regions			
	Sperm concentration (Mio/mL)		
Language region	0 – 15	15– 40	>40
**Latin (n = 44)**^a^	6 (13.6%)	16 (36.4%)	22 (50%)
**Non-Latin (n = 150)**^b^	27 (18%)	44 (29.3%)	79 (52.7%)

B) Area of residence			
	Sperm concentration (Mio/mL)		
Settlement type	0 – 15	15 – 40	>40
**Urban and Suburban (n = 146)**	25 (17.1%)	44 (30.1%)	77 (52.7%)
**Rural (n = 48)**	8 (16.7%)	16 (33.3%)	24 (50%)

a “Latin” includes the French-speaking (n = 35), Italian-speaking (n = 8), and Romansh-speaking (n = 1) regions.

b “Non-Latin” includes the German-speaking region (n = 150).

The Cochrane-Armitage trend test showed no evident trend in sperm concentration distribution across language regions (p = 0.52) and area of residence ((Sub)urban vs. Rural) (p = 0.69).

## 4 Discussion

This study analyzed sperm quality among young Swiss army recruits, comparing data from two cohorts: men recruited in the study of Rahban between 2005 and 2017 (Group 1) and military recruits evaluated in 2021 as part of the LoCoMo study (Group 2). By examining differences over time, hormonal profiles and geographical factors, this research enhances our understanding of male fertility within a single-country context.

### 4.1 Main outcomes

Comparing the 5th to 95th percentiles of the parameters, the study identified no further decline of sperm quality parameters. There was an increase in the proportion of men with at least one parameter below the WHO threshold, which is associated with a longer time to pregnancy [4]. No evidence for differences in sperm concentration were observed across language regions or settlement types. The interpretation of linguistic and urban versus rural differences in sperm concentration is constrained by the disproportionality of cases and the small sample size.

### 4.2 Reproductive hormones

The study identified evidence for hormonal differences among the three sperm concentration groups divided by WHO tresholds. This result supports the findings of other studies, such as a 2017 study that reported a negative correlation between FSH and semen parameters, as well as between LH and total sperm count and normal sperm morphology [29]. Elevated levels of LH and FSH were observed in individuals with lower sperm concentrations, indicating compensatory mechanisms for impaired spermatogenesis [30]. Testosterone levels did not differ between the groups.

### 4.3 Sperm quality over time: comparison of group 1 (Rahban) and group 2 (LoCoMo)

The findings revealed that sperm quality parameters among young Swiss men have not changed significantly. Semen volume, total sperm count, sperm concentration, progressive sperm motility, and sperm morphology remained relatively stable between 2005 and 2017 (18) and 2021 [19]. On the other hand, a comparison of the two studies showed that fewer young men had at least one sperm parameter below the WHO threshold (Group 1 (Rahban) 62% vs. Group 2 (LoCoMo) 41%).

This discrepancy may be attributed to differences in study size, laboratory differences, participant selection, or environmental factors.

### 4.4 COVID-19

For the recent COVID-19 subgroup, no significance testing could be performed; however, a comparison of the median values shows that sperm concentration and total sperm count were the lowest, which may suggest that COVID-19 could have had a transient impact on sperm quality. This negative impact was however short-lived as shown in the group who had had COVID-19 more than 6-months prior to testing. This was also shown by Donders et al. who found that sperm quality was most severely and negatively impacted during the first month after COVID-19 infection but that parameters returned to normal two months or later post infection [31,32].

### 4.5 Geographic variations

The geographical analysis of sperm concentration groups revealed no evidence for differences across Switzerland's language regions and areas of residence. This finding contrasts with studies from other countries where urbanization and agricultural exposure have been linked to variations in sperm quality [15,16].

## 5 Limitations and strengths

The representativeness of the study Group 2 (LoCoMo) is affected by selection bias, as participants were required to travel to Zurich for data collection. This led to a higher proportion of German-speaking soldiers. In addition, the small sample size of Group 2 (LoCoMo) limits the reliability of the findings and makes it difficult to compare with Group 1 (Rahban), which had a 10 fold larger sample size. However, comparisons with Rahban's study should be interpreted critically, as Group 1 (Rahban) included only a small sample of men between 2005 and 2017 (2523 out of 92,274). The limitations of Group 2 (LoCoMo) also affected specific analyses of geographical differences and further stratification, resulting in disproportional recruitment from certain areas.

In contrast of Group 1 (Rahban), Group 2 (LoCoMo) included only active military soldiers after recruitment, thus males that had already successfully passed recruitment, thus had a certain physical fitness and were considered generally healthy; while Group 1 (Rahban) included males who had not undergone medical screening during recruitment. Thus Group 2 (LoCoMo) potentially is biased towards more healthy and physically fit males when compared to Group 1 (Rahban). Since Rahban et al. did not report data on physical fitness, it was not possible to further assess this potential bias.

Despite these limitations, the study boasts several significant strengths. Data collection occurred over a period of less than a year, ensuring a robust and up-to-date, cross-sectional, representation of young Swiss men. Through the selection of recruits from the Swiss military, the LoCoMo study achieved a homogeneous cohort of men of comparable age and good general health. Additionally, the use of postal codes allowed for detailed geographical analysis, enabling an in-depth investigation of (sub)urban-rural and linguistic variations in sperm quality. Notably, in Group 2 (LoCoMo), hormonal levels could also be analyzed, an aspect not assessed in Group 1 (Rahban).

## 6 Conclusion

No differences in sperm quality parameters in Group 2 (LoCoMo) was observed by comparing the 5th to 95th percentiles with the findings of Group 1, the Rahban's study from the period 2005 to 2017. Some evidence for poorer progressive motile sperm count was found in the subgroup of recent COVID-19 infections (≤180 days before testing). Then poorer sperm quality results in those who had recently had COVID -19 were not seen in those whose COVID-19 infection predated testing by more than 6 months.Additionally we could show an inverse trend between LH and FSH levels and sperm concentration in Group 2 (LoCoMo). No evidence for differences in sperm concentration of Group 2 (LoCoMo) in Latin and Non-Latin language regions were found, as well as in (Sub)urban and rural regions. Further studies with larger sample sizes are required to better stratify the geographical differences in sperm concentration. Moreover, to establish sex hormones as biomarkers of sperm quality, further research is necessary that integrates hormonal analysis with sperm parameters, as there are still many controversial findings in the literature [11].

## CRediT authorship contribution statement

**Sabine Köppen:** Writing – original draft, Visualisation, Methodology, Formal analysis. **Ulrike Held:** Statistical analysis, Review & editing. **Nejla Gültekin, Andreas Stettbacher, Zeno Stanga, Ismail I. Ülgür:** Review & editing. **Jeremy Werner Deuel:** Statistical analysis, Review & editing. **Patricia Schlagenhauf:** Methodolgy, Formal analysis, Review & editing, Supervision, Project administration, Funding acquisition, Conceptualization.

## Disclosure statement

None.

## Attestation statements

The subjects in this trial have not concomitantly been involved in other randomized trials.

Data regarding any of the subjects in the study has not been previously published unless specified.

Data will not be made available to the editors of the journal for review or query upon request.

## Data sharing statement

Because of data protection regulation, individual data cannot be shared directly by the authors. We can share details of the test battery, questionnaires used, and some deidentified pooled data. Data sharing inquiries can be directed to patricia.schlagenhauf@uzh.ch after publication of the paper.

## Funding

The Swiss Armed Forces.

## Declaration of competing interest

The authors declare that they have no known competing financial interests or personal relationships that could have appeared to influence the work reported in this paper.
