# Supplementary material for: Sperm quality parameters of Swiss army recruits pre- and peri-COVID-19: a cross-sectional, comparative analysis
Source: New Microbes New Infect. 2026 May 5;71:101761. doi: 10.1016/j.nmni.2026.101761 (PMC13202535; doi:10.1016/j.nmni.2026.101761)
Supplement: Multimedia component 1 [file mmc1.docx]

**Supplemental Table 1:** **Associated table of the values in Figure 2.**

Comparison of the groups and subgroups by listing the parameters with the respective medians and the 5^th^ to 95^th^ percentiles.

|  | **Group 1 (Rhaban) (n = 2523)** | **Group 2 (LoCoMo) (n = 194) ^a^** | **Subgroup: LoCoMo (non-recent / asymptomatic COVID-19 group) (n = 96) ^b^** | **Subgroup: LoCoMo (recent COVID-19 group) (n = 11) ^c^** | **Subgroup: LoCoMo control group (n = 87) ^d^** |
| --- | --- | --- | --- | --- | --- |
| Ejaculation abstinence (days) | 2.8 (1.5⎼6.8) | 4 (2⎼7) | 4 (2⎼7) | 4 (2.5⎼6) | 3 (2⎼6) |
| Volume (mL) | 2.8 (1.0⎼5.6) | 3 (1.2⎼6.5) | 3 (1.2⎼6.6) | 2.5 (1.4⎼6.3) | 2.8 (1.4⎼6) |
| Sperm concentration (Mio/mL) | 47 (3.5⎼178) | 45 (4⎼100.7) | 44 (4.9⎼108) | 33 (2.5⎼73.5) | 50 (4.3⎼98.7) |
| Total sperm count (Mio) | 128 (7.7⎼524.5) | 120 (10.8⎼358.4) | 134 (13⎼365.8) | 75.9 (8.2⎼145.6) | 119 (14.2⎼355.5) |
| Motile sperm (%) | 53 (18.2⎼83.4) | 46 (18⎼71.1) | 47 (20.5⎼76.2) | 48 (10⎼57.5) | 45 (17.2⎼68.1) |
| Normal morphology (%) | 4 (0⎼17) | 10 (2⎼29.3) | 9 (2⎼29.8) | 9 (2.5⎼24.5) | 11 (2⎼27.2) |

^a^ total population (all subgroups together)

^b^ non-recent COVID-19 group (>180 days since positive COVID-19 PCR test) plus

asymptomatic COVID-19 group (serologically positive but with no symptoms)

^c^ recent COVID-19 group (≤180 days since positive COVID-19 PCR test)

^d^ control group (serologically negative)
